# Supplementary material for: Variegated overexpression of chromosome 21 genes reveals molecular and immune subtypes of Down syndrome
Source: Nat Commun. 2024 Jun 28;15:5473. doi: 10.1038/s41467-024-49781-1 (PMC11213896; doi:10.1038/s41467-024-49781-1)
Supplement: Supplementary file 1 — Supplementary Information [file 41467_2024_49781_MOESM1_ESM.pdf]

Supplementary Information for:

Variegated overexpression of chromosome 21 genes reveals molecular and immune subtypes of Down syndrome

Micah G. Donovan[1†], Neetha P. Eduthan[1†], Keith P. Smith[1], Eleanor C. Britton[1], Hannah R. Lyford[1], Paula Araya[1], Ross E. Granrath[1], Katherine A. Waugh[1,2], Belinda Enriquez-Estrada[1], Angela L. Rachubinski[1,3], Kelly D. Sullivan[1,4], Matthew D. Galbraith[1,2*], Joaquin M. Espinosa[1,2*]

Affiliations:

[1]Linda Crnic Institute for Down Syndrome, University of Colorado Anschutz Medical Campus; Aurora, USA.

[2]Department of Pharmacology, University of Colorado Anschutz Medical Campus; Aurora, USA.

[3]Department of Pediatrics, Section of Developmental Pediatrics, University of Colorado Anschutz Medical Campus; Aurora, USA.

[4]Department of Pediatrics, Section of Developmental Biology, University of Colorado Anschutz Medical Campus; Aurora, USA.

*Correspondence: joaquin.espinosa@cuanschutz.edu / matthew.galbraith@cuanschutz.edu

[†]These authors contributed equally to this work

Table of Contents

Supplementary Fig. 1 3
Supplementary Fig. 2 5
Supplementary Fig. 3 7
Supplementary Fig. 4 9
Supplementary Fig. 5 11
Supplementary Fig. 6 13
Supplementary Fig. 7 15
Supplementary Fig. 8 17

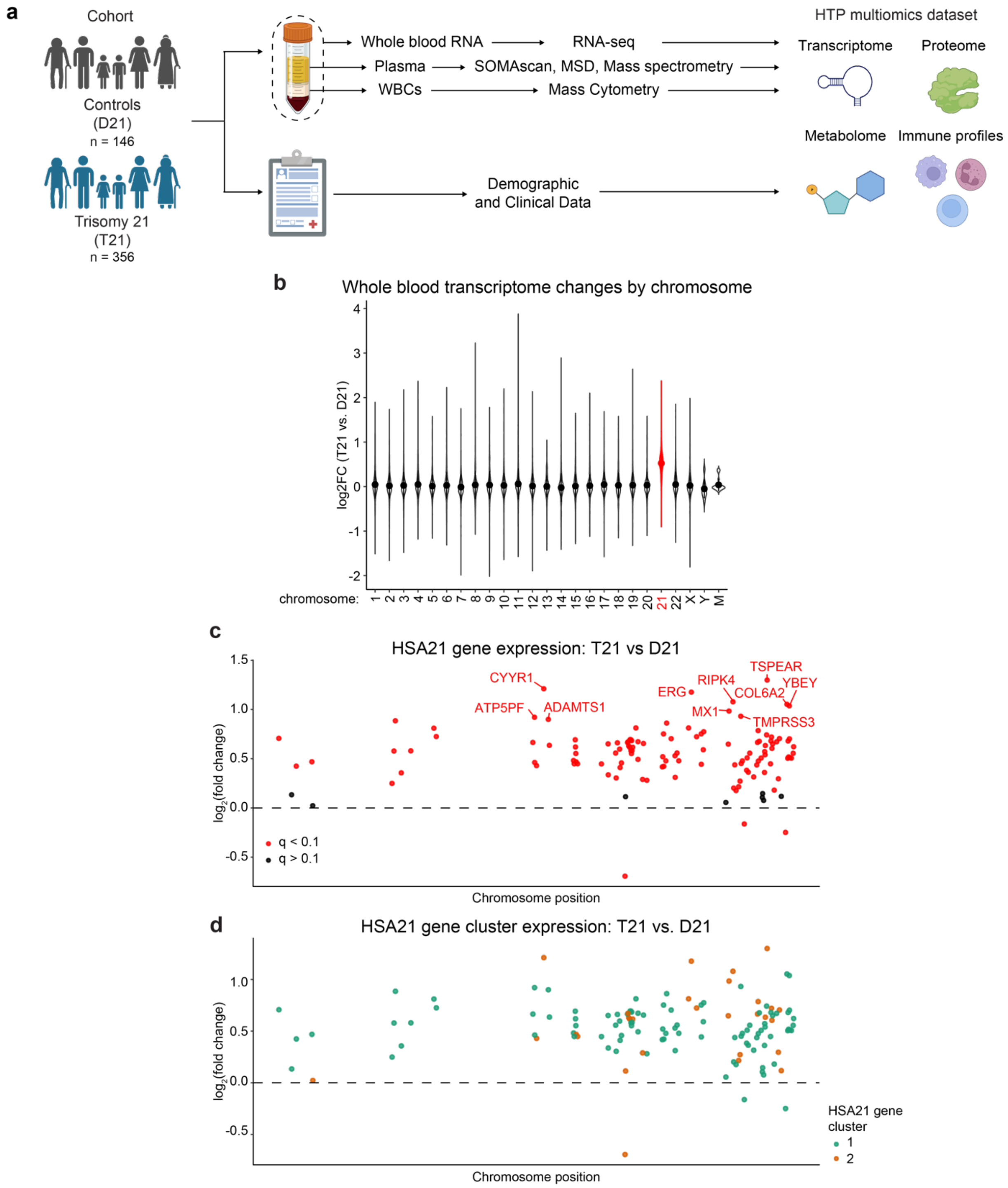

Supplementary Fig. 1 | Individuals with Down syndrome show variegated overexpression of genes encoded on chromosome 21.

a Schematic of Human Trisome Project biospecimen collection and analytical pipeline. **b** Violin plot showing relative expression derived from DESeq2 for all genes, separated by chromosome along the X-axis, in individuals with T21 (n = 304) compared to euploid controls (n = 96). **c** Point plot showing differential expression of HSA21 genes in individuals with T21 (n = 304) relative to euploid controls (D21, n = 96). Differential expression was determined by DESeq2 with a significance cutoff of $q < 0.1$ after adjustment using the Benjamini-Hochberg method. Genes are arranged on X-axis by chromosomal location on HSA21. **d** Point plot showing results in (**c**) with colors indicating HSA21 gene clusters (cluster 1, teal; cluster 2, orange). Supplementary Figure 1/panel a created with BioRender.com released under a Creative Commons Attribution-NonCommercial-NoDerivs 4.0 International license.

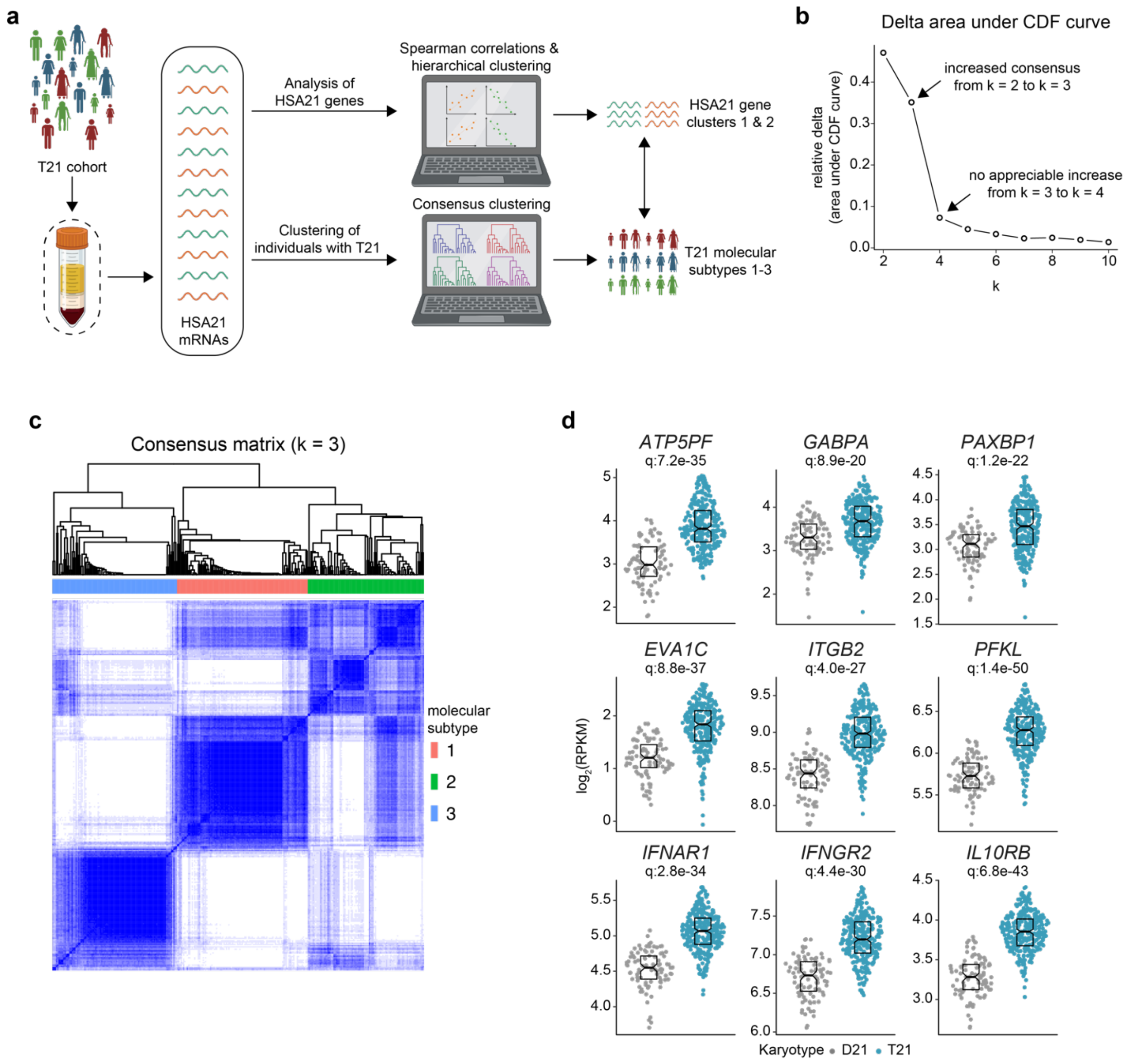

Supplementary Fig. 2 | Variable chromosome 21 gene expression distinguishes molecular subtypes in Down syndrome.

a Whole blood RNA-seq data from individuals with trisomy 21 (T21) were analyzed to identify co-expression clusters of chromosome 21 (HSA21) genes and to classify molecular subtypes within the T21 group based on HSA21 gene expression. Unsupervised hierarchical clustering, utilizing Spearman correlations of RPKMs, defined the HSA21 gene clusters (cluster 1, teal; cluster 2; orange). The molecular subtypes were delineated through consensus clustering of z-

scored RPKM data, calculated from the mean and standard deviation of HSA21 gene expression data exclusively within the T21 cohort. **b** Line plot showing area under cumulative distribution function (CDF) curves for relative changes in number of k clusters from consensus clustering analysis of T21 (n = 304) HSA21 gene expression. **c** Heatmap showing clustering of HSA21 genes in individuals with T21 (n = 304) based on a three-group clustering solution from consensus cluster plus analysis. Colors denote molecular subtype (MS) classification (MS1, red; MS2, green; MS3, blue). **d** Sina plots showing expression of HSA21 genes (from top left to bottom right) *ATP5PF, GABPA, PAXBP1, EVA1C, ITGB2, PFKL, IFNAR1, IFNGR2, and IL10RB*, in individuals with T21 (n = 304, teal) compared to euploid controls (D21, n = 96, gray). q-values are derived from DESeq2 comparisons to D21 and adjusted using the Benjamini-Hochberg method. Boxes represent interquartile ranges and medians, with notches approximating 95% confidence intervals. Supplementary Figure 2/panel a created with BioRender.com released under a Creative Commons Attribution-NonCommercial-NoDerivs 4.0 International license.

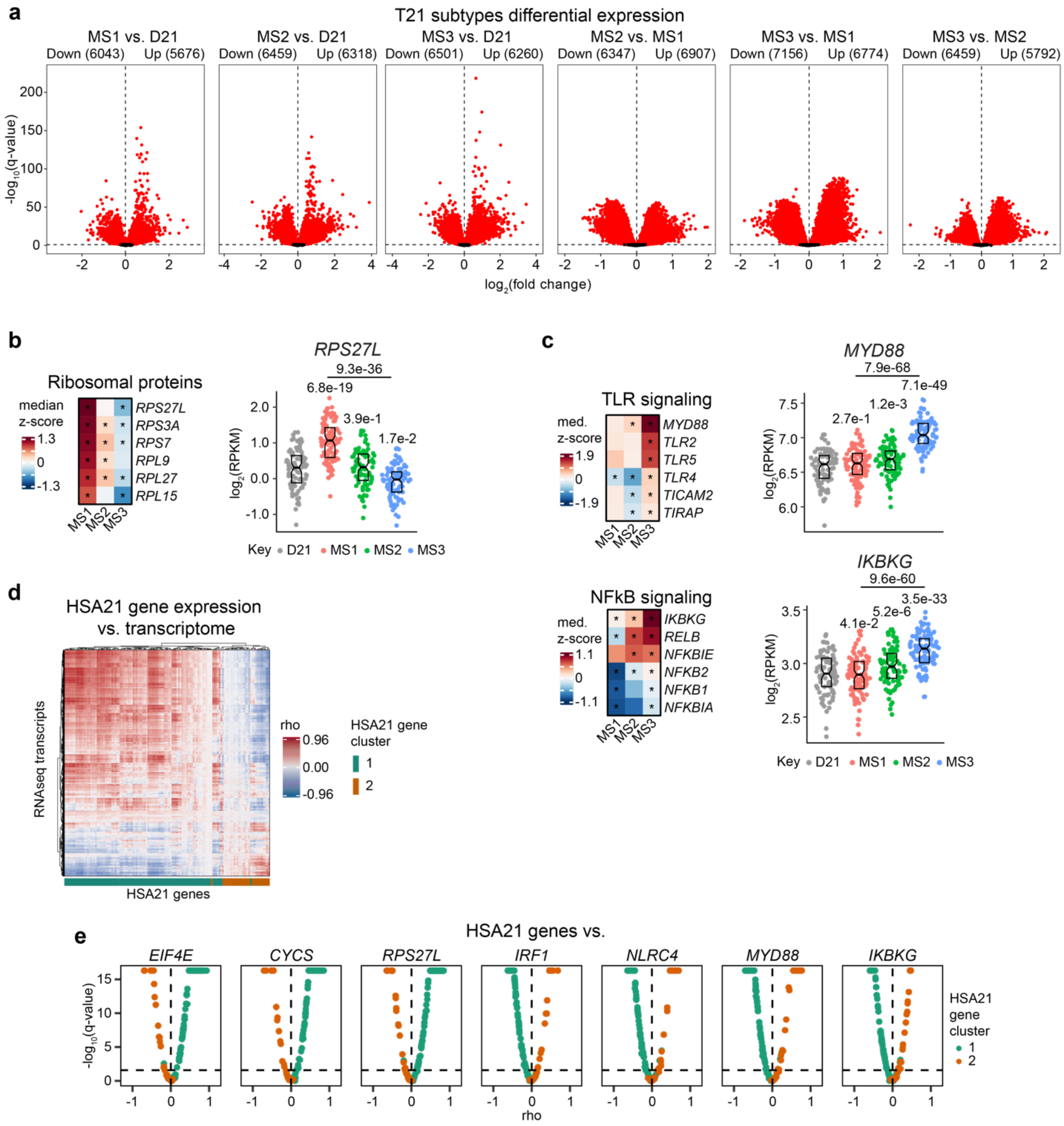

Supplementary Fig. 3 | Molecular subtypes of Down syndrome are distinguished by distinct transcriptomic landscapes.

a Volcano plots depicting results from DESeq2 analysis comparing molecular subtypes (MS1, n = 107; MS2, n = 95; MS3, n = 102) to euploid controls (panels 1-3, n = 96) and each other (panels 4-6). **b-c** Heatmaps display median z-scores for representative genes in MS1 (n=107,

red), MS2 (n=95, green), and MS3 (n=102, blue) calculated relative to D21 (n=96, gray), with asterisks denoting q < 0.1 from DESeq2 after Benjamini-Hochberg adjustment. Sina plots illustrate gene expression across groups. q-values, derived from DESeq2 and adjusted using the Benjamini-Hochberg method, are displayed above individual data swarms for comparisons to D21 and above lines for MS3 vs. MS1. Boxes represent interquartile ranges and medians, with notches approximating 95% confidence intervals. **d** Heatmap showing Spearman correlations for HSA21 gene expression vs. expression of non-HSA21 genes. Dendrograms indicate results from unsupervised hierarchical clustering. HSA21 gene clusters are indicated by colors denoted in figure panel (cluster 1, teal; cluster 2, orange). **e** Volcano plots showing Spearman correlations of non-HSA21 genes overexpressed in MS1 (*EIF4E, CYCS, RPS27L*) or MS3 (*IRF1, NLRC4, MYD88, IKBKG*) vs. HSA21 cluster 1 (teal) and cluster 2 (orange) genes in individuals with T21 (n = 304). Dashed line indicates q = 1.

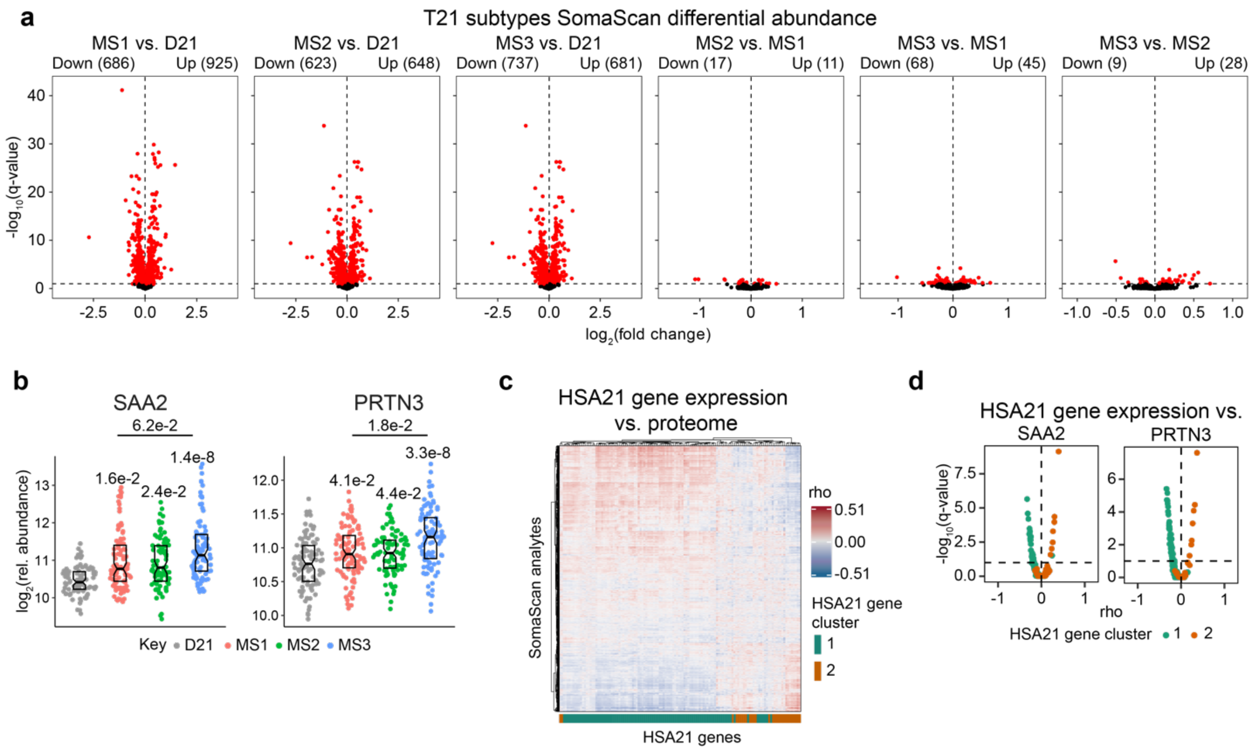

Supplementary Fig. 4 | Plasma proteomics reveals varied immune and inflammatory dysregulation across subtypes of Down syndrome.

a Volcano plots depicting results from linear regression analyses comparing molecular subtypes (MS1, n = 107; MS2, n = 95; MS3, n = 102) to euploid (D21) controls (panels 1-3, n = 103) and each other (panels 4-6). **b** Sina plots (right) showing expression of exemplary genes across in D21, n = 103, gray), MS1 (n = 107, red), MS2 (n = 95, green), and MS3 (n = 102, blue). q-values, derived from linear regressions and adjusted using the Benjamini-Hochberg method, are displayed above individual data swarms for comparisons to D21 and above lines for MS3 vs. MS1. Boxes represent interquartile ranges and medians, with notches approximating 95% confidence intervals. **c** Heatmap showing Spearman correlations for HSA21 gene expression vs. proteins detected in SomaScan analysis. Dendrograms indicate results from unsupervised hierarchical clustering. Cluster 1 (teal) and 2 (orange) HSA21 genes are indicated by colors denoted in figure panel. **d** Volcano plot showing Spearman correlations between levels SAA2

and PRTN3 vs. expression of cluster 1 (teal) and cluster 2 (orange) HSA21 genes in individuals with T21 (n = 304). Dashed line indicates q = 0.1.

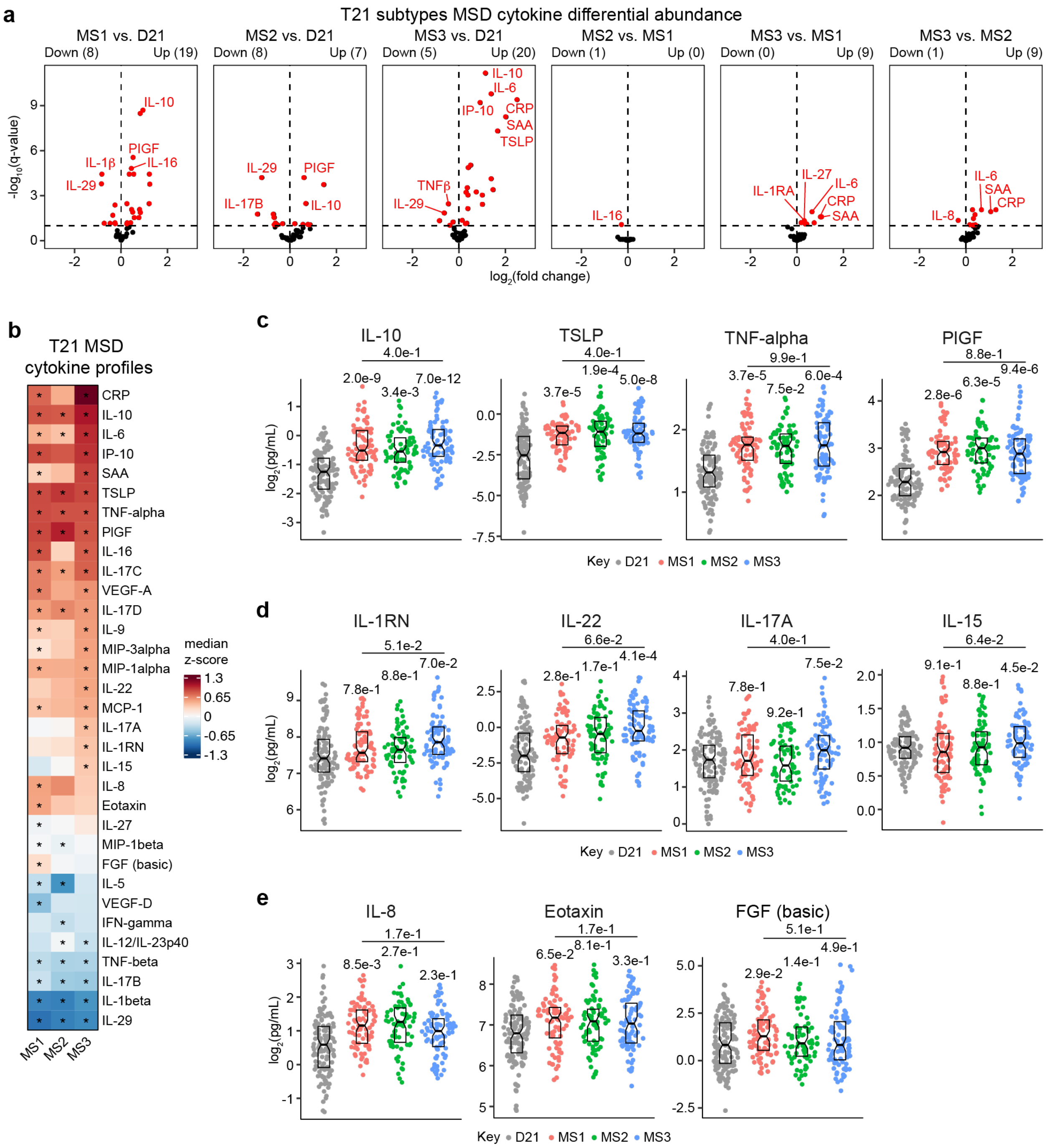

Supplementary Fig. 5 | Molecular subtypes associate with distinct inflammatory milieus.

a Volcano plots depicting results from linear regression analyses of MSD inflammatory marker data comparing molecular subtypes (MS1, n = 87; MS2, n = 75; MS3, n = 87) to euploid controls (panels 1-3, n = 131) and each other (panels 4-6). **b** Heatmap showing median z scores relative

to euploid controls (D21, n = 131) of MSD inflammatory markers in MS1 (n = 87), MS2 (n = 75) and MS3 (n = 87). Asterisks indicate significance ($q < 0.1$) from linear regressions vs. D21 after Benjamini-Hochberg adjustment for multiple hypotheses. **c-e** Sina plots showing levels of example immune markers across in D21 (n = 131, gray), MS1 (n = 87, red), MS2 (n = 75, green), and MS3 (n = 87, blue). q-values, derived from linear regressions and adjusted using the Benjamini-Hochberg method, are displayed above individual data swarms for comparisons to D21 and above lines for MS3 vs. MS1. Boxes represent interquartile ranges and medians, with notches approximating 95% confidence intervals.

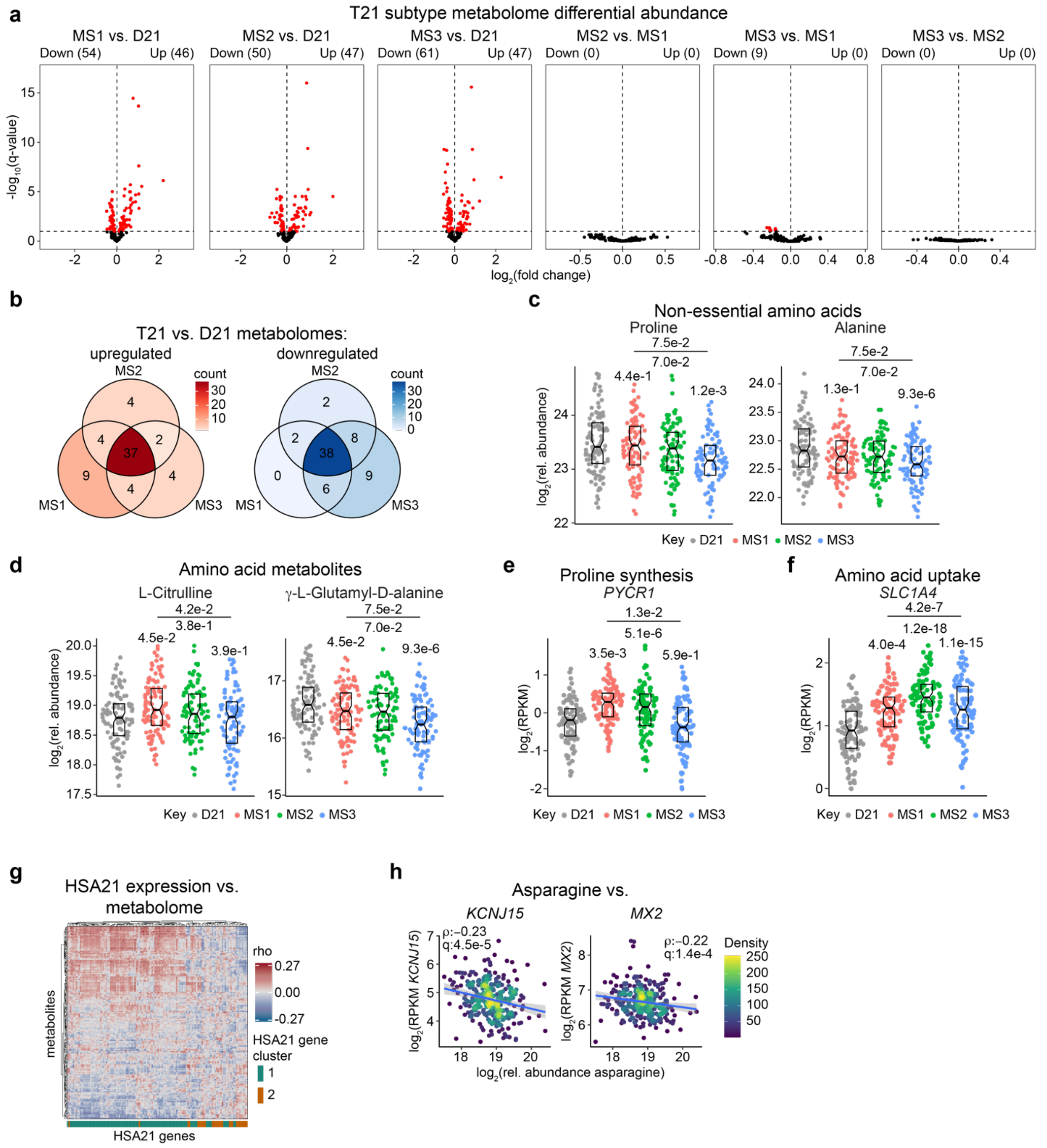

Supplementary Fig. 6 | Plasma metabolomics reveals depletion of amino acids associates with MS3.

a Volcano plots depicting results from linear regression analyses of LC-MS metabolomics data comparing molecular subtypes (MS1, n = 107; MS2, n = 95; MS3, n = 102) to euploid controls

(panels 1-3, n = 103) and each other (panels 4-6). **b** Overlapping differentially abundant metabolites identified by linear regressions comparing MS1 (n = 107), MS2 (n = 95) and MS3 (n = 102) against D21 (n = 102). **c-d** Sina plots showing levels of non-essential amino acids (**c**) and amino acid metabolites (**d**) across in D21 (n = 102, gray), MS1 (n = 107, red), MS2 (n = 95, green) and MS3 (n = 102, blue). q-values, derived from linear regressions and adjusted using the Benjamini-Hochberg method, are displayed above individual data swarms for comparisons to D21 and above lines for MS3 vs. MS1. Boxes represent interquartile ranges and medians, with notches approximating 95% confidence intervals. **e-f** Sina plots showing gene expression for amino acid synthesis gene *PYCR1* (**e**) and transporter *SLC1A4* (**f**) in euploid controls (D21, n = 96, gray), MS1 (n = 107, red), MS2 (n = 95, green) and MS3 (n = 102, blue). Boxes represent interquartile ranges and medians, with notches approximating 95% confidence intervals. q-values, derived from DESeq2 and adjusted using the Benjamini-Hochberg method, are displayed above individual data swarms for comparisons to D21 and above lines for MS3 vs. MS1. **g** Heatmap showing Spearman correlations for HSA21 gene expression vs. metabolites detected in LC-MS analysis. Dendrograms indicate results from unsupervised hierarchical clustering. Cluster 1 (teal) and 2 (orange) HSA21 genes are indicated by colors denoted in the figure panel. **h** Scatter plots depicting relationship between levels of asparagine versus expression of cluster 2 HSA21 genes *KCNJ15* and *MX2*. *rho* and q-values (Benjamini-Hochberg adjusted p-values) for Spearman correlation are denoted. Points are colored by density; blue lines represent the fitted values from linear regressions, with 95% confidence intervals in grey.

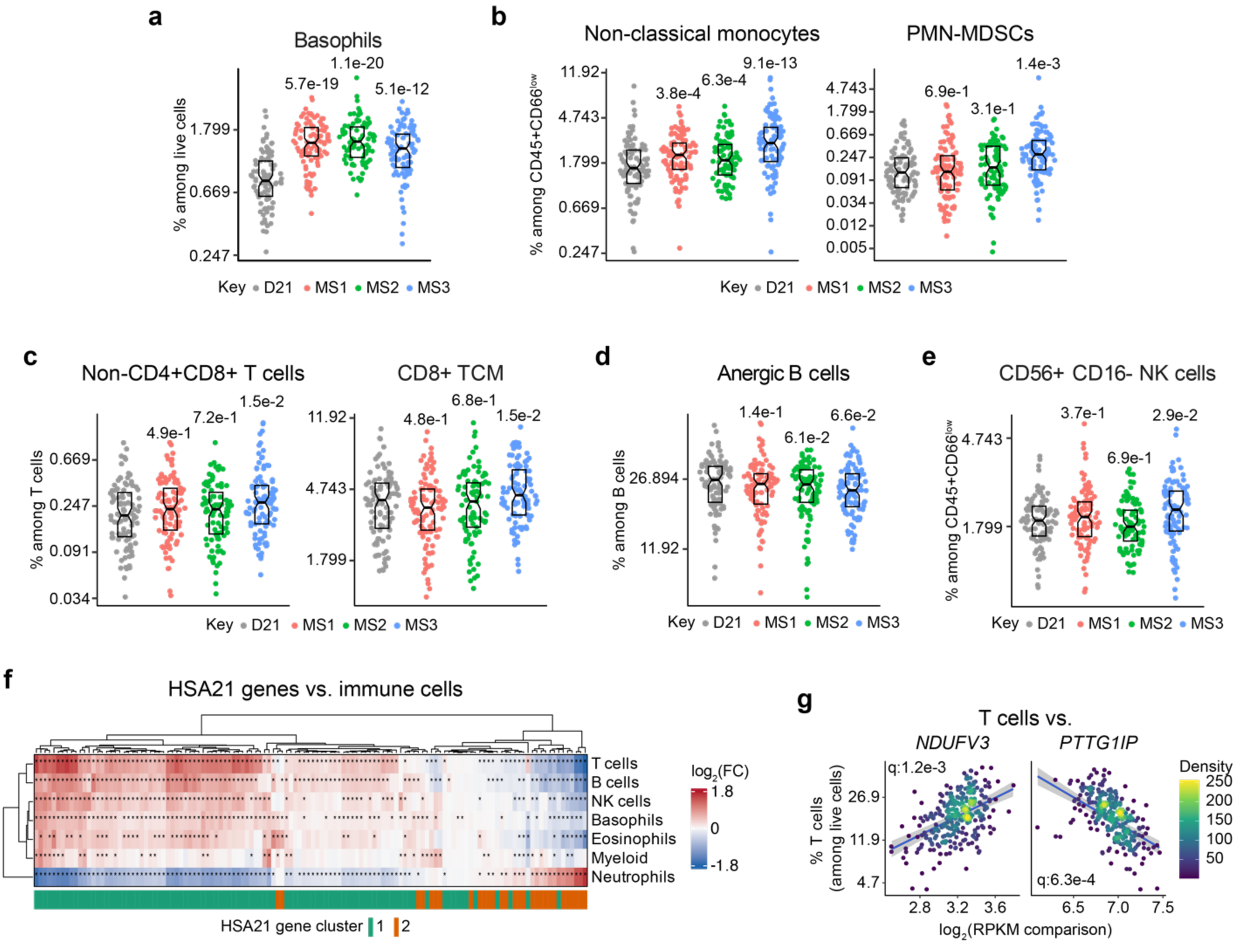

Supplementary Fig. 7 | The molecular subtypes of Down syndrome present unique immune cell landscapes.

a-e Sina plots showing levels of basophils (**a**), non-classical monocytes and PMN-MDSCs (**b**), non-CD4+/CD8+ and CD8+ T cells (**c**), anergic B cells (**d**), and CD56+/CD16- NK cells (**e**) in MS1 (n = 98, red), MS2 (n = 90, green), MS3 (n = 96, blue) compared to euploid controls (D21, n = 90, gray). q-values, derived from beta regressions and adjusted using the Benjamini-Hochberg method, are displayed above individual data swarms for comparisons to D21. Boxes represent interquartile ranges and medians, with notches approximating 95% confidence intervals. **f** Heatmap depicting transformed fold-change values from beta-regressions comparing expression of HSA21 genes to abundance of immune cell types from CyTOF analysis.

Regressions were performed in individuals with T21 only (n = 284). Asterisks denote significance q < 0.1 after adjustment by Benjamini-Hochberg multiple hypothesis correction. Dendrograms indicate results of unsupervised hierarchical clustering. Cluster 1 (teal) and 2 (orange) HSA21 genes are indicated by colors denoted in the figure panel. **g** Scatter plots depicting relationship between total T cells vs. expression of cluster 1 (*NDUFV3*) and cluster 2 (*PTTG1IP*) HSA21 genes. Points are colored by density; blue lines represent the fitted values from beta regressions, with 95% confidence intervals in grey. Significance is defined as q < 0.1 after multiple hypothesis correction using the Benjamini-Hochberg method.

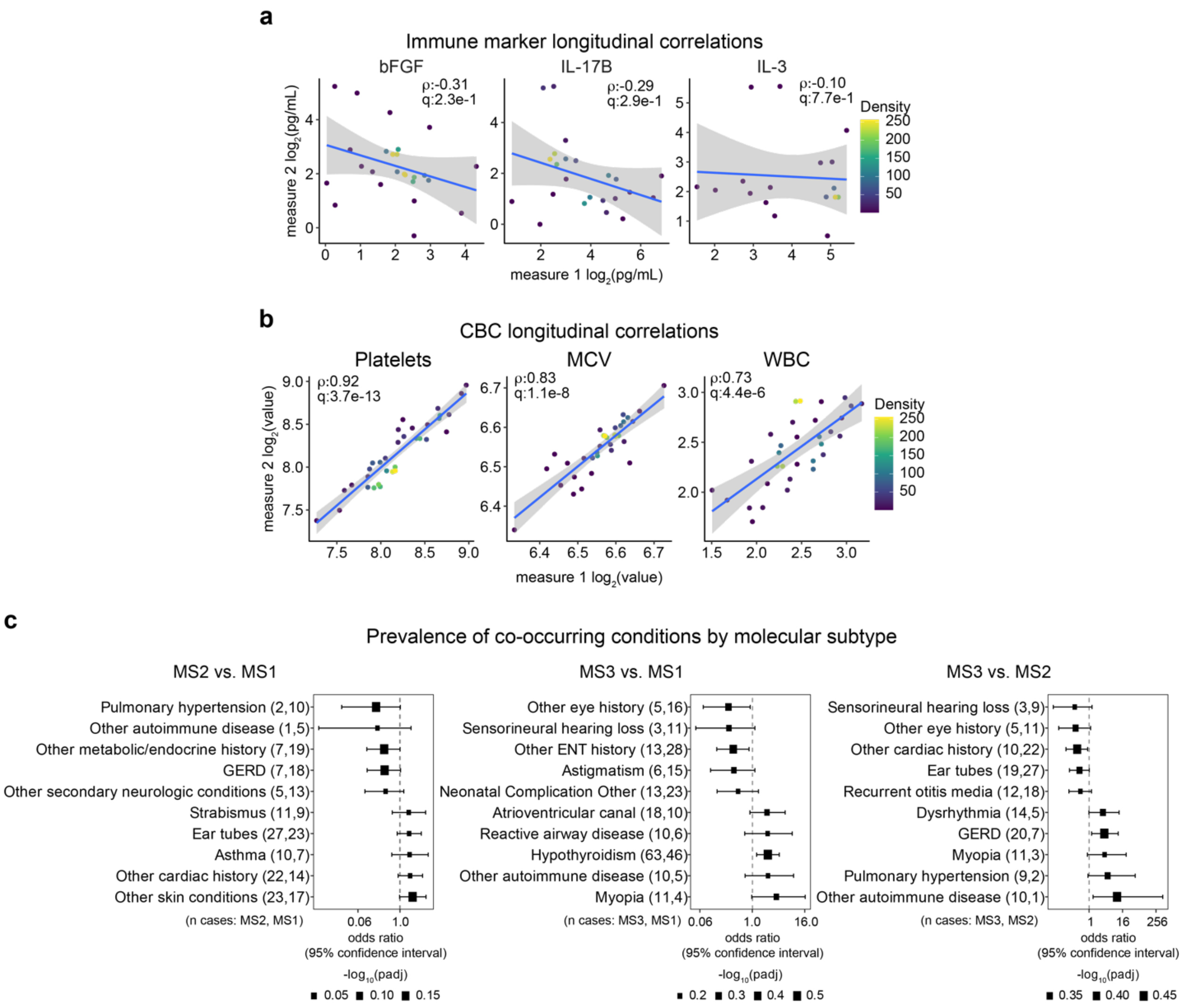

Supplementary Fig. 8 | Temporal stability and clinical detection of molecular subtypes of Down syndrome.

a-b Scatter plots depicting correlations for levels of inflammatory markers (**a,** n = 25) and CBC parameters (**b,** n = 33) between longitudinal visits in individuals with T21. *rho* and q-values (Benjamini-Hochberg adjusted p-values) for Spearman correlation are denoted. Points are colored by density; blue lines represent the fitted values from linear regressions, with 95% confidence intervals in grey. **c** Forest plots depict pairwise comparisons between trisomy 21 (T21) molecular subtypes (MS) regarding the overrepresentation of cases versus controls for co-occurring conditions associated with Down syndrome (DS). For each pairwise comparison,

the plots display the five strongest positive and five strongest negative associations. Fisher's exact test was employed to assess statistical differences in overrepresentation. Square point sizes are inversely proportional to the q-values, indicating significance levels, and error bars represent 95% confidence intervals. The number of confirmed cases for each subtype is indicated in parentheses.
