# Supplementary material for: Variegated overexpression of chromosome 21 genes reveals molecular and immune subtypes of Down syndrome
Source: Nat Commun. 2024 Jun 28;15:5473. doi: 10.1038/s41467-024-49781-1 (PMC11213896; doi:10.1038/s41467-024-49781-1)
Supplement: Supplementary file 3 — Description of Additional Supplementary Files [file 41467_2024_49781_MOESM3_ESM.pdf]

Description of Additional Supplementary Files

File Name: Supplementary Data 1
Description: **Whole blood chromosome 21 gene expression correlations in trisomy 21.**
a Results for Spearman correlations (rho) between expression of chromosome 21 genes from whole-blood RNAseq in individuals with trisomy 21 (T21).

File Name: Supplementary Data 2
Description: **Trisomy 21 molecular subtypes whole-blood transcriptome differential expression and pathway analysis. a** Clinical variables, including karyotype, molecular subtype (MS), sex, age, and BMI, for all participants in the RNA-seq analysis with matched plasma proteome and metabolome, and immune cell data. **b** Whole blood RNAseq DESeq2 results of HSA21 genes for trisomy 21 (T21) MS versus euploid controls (D21) and each other. **c** Whole blood RNAseq DESeq2 results of all genes for MS versus D21. **d** Ingenuity Pathway Analysis (IPA) of canonical pathways among whole blood transcriptome data for all T21 and by individual MS. **e** IPA of upstream regulators among whole blood transcriptome data for all T21 and by individual MS.

File Name: Supplementary Data 3
Description: **Trisomy 21 molecular subtypes plasma proteomics differential abundance and pathway analysis. a** Plasma proteomics (SomaScan) linear regression results for trisomy 21 (T21) molecular subtypes (MS) versus euploid controls (D21) and each other. **b** Ingenuity Pathway Analysis (IPA) of canonical pathways among plasma proteome data for all T21 and by individual MS. **c** IPA of diseases and biofunctions among plasma proteome data for all T21 and by individual MS.

File Name: Supplementary Data 4
Description: **Trisomy 21 molecular subtypes plasma inflammatory markers differential abundance. a** Plasma inflammatory markers (MSD) linear regression results for trisomy 21 (T21) molecular subtypes (MS) versus euploid controls (D21) and each other.

File Name: Supplementary Data 5
Description: **Trisomy 21 molecular subtypes plasma metabolomics differential abundance.**
a Plasma metabolomics (LCMS) linear regression results for trisomy 21 (T21) molecular subtypes (MS) versus euploid controls (D21) and each other.

File Name: Supplementary Data 6
Description: **Trisomy 21 molecular subtypes immune cell and complete blood count differential abundance. a** Beta regression results for cell subpopulation proportions measured by mass cytometry (CyTOF) for trisomy 21 (T21) molecular subtypes (MS) versus euploid controls (D21). **b** Complete blood count (CBC) linear regression results for MS versus D21.

File Name: Supplementary Data 7
Description: **Temporal stability of plasma inflammatory markers and complete blood counts in trisomy 21. a** Results for Spearman correlations (rho) between levels of plasma inflammatory markers (MSD) across longitudinal blood draws. **b** Results for Spearman

correlations (rho) between complete blood count (CBC) parameters across longitudinal blood draws.

File Name: Supplementary Data 8
Description: **Key antibodies and reagents used for mass cytometry analysis. a** List of antibodies for cell-specific antigens. **b** List of metal isotypes for labeling.
