# Supplementary material for: Variegated overexpression of chromosome 21 genes reveals molecular and immune subtypes of Down syndrome
Source: Nat Commun. 2024 Jun 28;15:5473. doi: 10.1038/s41467-024-49781-1 (PMC11213896; doi:10.1038/s41467-024-49781-1)
Supplement: Supplementary file 12 — Reporting Summary [file 41467_2024_49781_MOESM12_ESM.pdf]

nature portfolio

Corresponding author(s): Joaquin Espinosa & Matthew Galbraith

Last updated by author(s): Mar 13, 2024

Reporting Summary

Nature Portfolio wishes to improve the reproducibility of the work that we publish. This form provides structure for consistency and transparency in reporting. For further information on Nature Portfolio policies, see our Editorial Policies and the Editorial Policy Checklist.

Statistics

For all statistical analyses, confirm that the following items are present in the figure legend, table legend, main text, or Methods section.

n/a	Confirmed	
☐	☒	The exact sample size (*n*) for each experimental group/condition, given as a discrete number and unit of measurement
☐	☒	A statement on whether measurements were taken from distinct samples or whether the same sample was measured repeatedly
☐	☒	The statistical test(s) used AND whether they are one- or two-sided<br>*Only common tests should be described solely by name; describe more complex techniques in the Methods section.*
☐	☒	A description of all covariates tested
☐	☒	A description of any assumptions or corrections, such as tests of normality and adjustment for multiple comparisons
☐	☒	A full description of the statistical parameters including central tendency (e.g. means) or other basic estimates (e.g. regression coefficient) AND variation (e.g. standard deviation) or associated estimates of uncertainty (e.g. confidence intervals)
☐	☒	For null hypothesis testing, the test statistic (e.g. *F*, *t*, *r*) with confidence intervals, effect sizes, degrees of freedom and *P* value noted<br>*Give P values as exact values whenever suitable.*
☒	☐	For Bayesian analysis, information on the choice of priors and Markov chain Monte Carlo settings
☒	☐	For hierarchical and complex designs, identification of the appropriate level for tests and full reporting of outcomes
☐	☒	Estimates of effect sizes (e.g. Cohen's *d*, Pearson's *r*), indicating how they were calculated

Our web collection on statistics for biologists contains articles on many of the points above.

Software and code

Policy information about availability of computer code

Data collection	RNA-seq data were collected with an Illumina NovaSeq 6000 instrument. Inflammatory marker data were collected with MESO QuickPlex SQ 120 instrument. Plasma proteomics data were collected by SomaLogic using the SomaScan platform. Mass cytometry data were collected with a Helios mass cytometer (Fluidigm). Mass spectrometry data were collected with a Vanquish UHPLC coupled online to a Q Exactive high resolution mass spectrometer (ThermoFisher Scientific). Complete blood count data were collected with an AcT 10 hematology analyzer (Beckman Coulter).
Data analysis	No new software was developed during this study. All data analysis was carried out using existing software as described in the Online Methods for each specific experiment. Software used in this study includes R v4.0+, R studio v2022.12.0, Bioconductor v3.16+, QIAGEN IPA version 01-22-01 (QIAGEN Inc.), FASTQC v0.11.5, FastQ Screen v0.11.0, BBTools v37.99, ea-utils v1.05, HISAT2 v2.1.0, Samtools v1.5, HTSeq-count v0.6.1, Matlab (v9.12) CellEngine (CellCarta, accessed 2022), Adobe Illustrator v25.1, Microsoft Word v16.78, Microsoft Excel v16.82, and EndNote v9.3.3. R packages used in this study include ggplot2 v3.4.4, ggforce v0.4.1, ComplexHeatmap v2.16.0, tidyHeatmap v1.8.1, ConsensusClusterPlus v1.64.0, fgsea v1.14.0, Hmisc v5.1.1, DESeq2 v1.40.2, limma v3.56.2, SomaDataIO v3.1.0, flowCore v2.0.1, flowVS v1.34.0, CATALYST v1.12.2, tidySingleCellExperiment v1.3.3, MEM v3, betareg v3.1.1-4, and datawizard v0.9.0.

For manuscripts utilizing custom algorithms or software that are central to the research but not yet described in published literature, software must be made available to editors and reviewers. We strongly encourage code deposition in a community repository (e.g. GitHub). See the Nature Portfolio guidelines for submitting code & software for further information.

Data

Policy information about availability of data

All manuscripts must include a data availability statement. This statement should provide the following information, where applicable:

- Accession codes, unique identifiers, or web links for publicly available datasets
- A description of any restrictions on data availability
- For clinical datasets or third party data, please ensure that the statement adheres to our policy

All data needed to evaluate the conclusions in the paper are present in the paper and/or the Supplementary Materials. The demographics and clinical data used in this study have been deposited on the Synapse data sharing platform under accession code syn31488784 (https://doi.org/10.7303/syn31488784). The whole blood RNA-seq data used in this study have been deposited on Synapse under accession code syn31488780 (https://doi.org/10.7303/syn31488780) and Gene Expression Omnibus under accession code GSE190125 (https://www.ncbi.nlm.nih.gov/geo/query/acc.cgi?acc=GSE190125). The MSD plasma proteomics data used in this study have been deposited on Synapse under accession code syn31475487 (https://doi.org/10.7303/syn31475487). The SomaScan plasma proteomics data used in this study have been deposited on Synapse under accession code syn31488781 (https://doi.org/10.7303/syn31488781). The plasma LC-MS metabolomics data used in this study have been deposited on Synapse under accession code syn31488782 (https://doi.org/10.7303/syn31488782) and Metabolomics Workbench under accession code ST002200 (http://dev.metabolomicsworkbench.org:22222/data/DRCCMetadata.php?Mode=Study&StudyID=ST002200&Access=UrlT3545). The immune cell mass cytometry data used in this study have been deposited on Synapse under accession code syn31488783 (https://doi.org/10.7303/syn31488783). The entire integrated multidimensional dataset used for this study has been deposited on the Synapse data sharing platform under accession code syn31481952 (https://doi.org/10.7303/syn31481952.5) and the INCLUDE Data Hub (https://portal.includedcc.org/). Please note that the INCLUDE Data Hub does not provide URLs for specific datasets. This is a registered access platform that requires users to create an account and request access to these data files. All data used in this study are also provided in Source Data files. Databases used in the generation of these results include (KEGG, https://www.genome.jp/kegg/), the Human Molecular Signatures Database (MSigDB, https://www.gsea-msigdb.org/gsea/msigdb/collections.jsp), the Genome Reference Consortium Human Build 38 (GRCh38, https://www.ncbi.nlm.nih.gov/grc/human), GENCODE Release 33 annotations (https://www.gencodegenes.org/human/release_33.html).

Research involving human participants, their data, or biological material

Policy information about studies with human participants or human data. See also policy information about sex, gender (identity/presentation), and sexual orientation and race, ethnicity and racism.

Reporting on sex and gender	This study reports analysis of datasets from a cohort of 185 euploid controls (males=70, females=115) and 304 individuals with Down syndrome (males=163, females=141). From this overall cohort, the study focuses primarily on a subset of participants with matched multiomics dataset (whole blood transcriptome, plasma proteome and metabolome, and immune cell mass cytometry data) from 400 research participants. 304 of these participants have Down syndrome, or trisomy 21 (T21), including 141 females and 163 males. The remaining 96 participants are euploid controls, consisting of 52 females and 44 males. The focus of this investigation was to define molecular subtypes (MS) of T21 based on chromosome 21 (HSA21) transcriptomic data and delineate molecular differences between these subtypes. Upon identifying these subtypes, we compared their ratios of males to females and observed no differences (MS1, n females = 50, n males = 57; MS2, n females = 43 , n males = 52 ; MS3, n females = 48 , n males = 54). Thus, our investigation focused solely on the comparing these subtypes to D21 and each other. Of note sex was used as a covariate when making comparisons between the MS and D21 and to each other.
Reporting on race, ethnicity, or other socially relevant groupings	This study does not report on any socially constructed or socially relevant categorization variables.
Population characteristics	This study reports analysis of datasets from a cohort of 185 euploid controls (males=70, females=115) and 356 individuals with Down syndrome (males=163, females=141). From this overall cohort, this study focuses on whole blood transcriptome, plasma proteome and metabolome, and immune cell mass cytometry data from 400 research participants, 304 with trisomy 21 (T21) and 96 euploid controls (D21). The T21 cohort was further stratified into 3 distinct molecular subtypes (MS). Our investigation focused on comparisons between MS versus D21 and each other. In addition to using sex, age was also used as a key-covariate when making these analytical comparisons. The median age and interquartile range for D21 is 27.6 years (14.4 - 38.9), MS1 is 22.9 years (13.3 - 33.2), MS2 is 24.4 years (17.2 - 30.4), and MS3 is 22.4 years (16.8 - 31.4).
Recruitment	Recruitment into the Crnic Institute Human Trisome Project (HTP) took place at the University of Colorado Anschutz Medical Campus in Aurora, Colorado, USA, as well as multiple conferences in the USA. The study was promoted through scientific presentations at community conferences, IRB-approved flyers, the Human Trisome Project website (www.trisome.org), websites from affiliated organizations (e.g. the Global Down Syndrome Foundation), the DS-Connect registry, and social media. Participants received US$100 compensation per blood draw. Procedures were performed in accordance with IRB guidelines and regulations. Given the focus on recruitment of individuals with Down syndrome, a self-selection bias is unlikely, as recruitment is based on karyotype, which is confirmed from review of electronic health records.
Ethics oversight	Research participants were enrolled to the Crnic Institute Human Trisome Project Biobank (HTP) under a study protocol approved by the Colorado Multiple Institutional Review Board (IRB; COMIRB #15-2170). Procedures were performed in accordance with COMIRB guidelines and regulations. Written informed consent was obtained from participants who were cognitively able or by guardians of each participant. The study was conducted in accordance with the Declaration of Helsinki.

Note that full information on the approval of the study protocol must also be provided in the manuscript.

Field-specific reporting

Please select the one below that is the best fit for your research. If you are not sure, read the appropriate sections before making your selection.

☒ Life sciences ☐ Behavioural & social sciences ☐ Ecological, evolutionary & environmental sciences

For a reference copy of the document with all sections, see nature.com/documents/nr-reporting-summary-flat.pdf

Life sciences study design

All studies must disclose on these points even when the disclosure is negative.

Sample size	No sample size or power calculations were employed. This investigation used the entire available dataset.
Data exclusions	In comparative analyses and visualizations of whole blood RNA-seq, plasma SOMAscan proteomics, plasma LC-MS metabolics, plasma inflammatory markers, complete blood counts and immune cell population measurements, extreme outliers were classified per-karyotype (or molecular subtype) and per-analyte as measurements more than three times the interquartile range above the first and third quartiles, respectively, and excluded.
Replication	The focus of this investigation was to evaluate differences between individuals with trisomy 21 (T21) and euploid controls (D21). An additional focus was to investigate differences when comparing three molecular subtypes (MS1-3) of T21 (defined in the manuscript) versus D21 and each other. For RNA-seq the number of biological replicates for was as follows: D21 n=96, T21 n=304, MS1 n=107, MS2 n=95, MS3 n=102. For plasma proteome and metabolome: D21 n=103, T21 n=304, MS1 n=107, MS2 n=95, MS3 n=102. For MSD inflammatory markers: D21 n=131, T21 n=249, MS1 n=87, MS2 n=75, MS3 n=87. For mass cytometry immune cell measurements: D21 n=96, T21 n=284, MS1 n=98, MS2 n=90, MS3 n=96. For CBC measurements: D21 n=70, T21 n=91, MS1 n=39, MS2 n=19, MS3 n=33. For all datasets, except for MSD inflammatory markers, number of technical replicates was n=1. For, MSD inflammatory markers, the number of technical replicates was n=2 and the values used for these analyses were derived from the mean. All attempt at replication were successful.
Randomization	Randomization was not required for this retrospective study, which employs covariate adjustment for sex, age, and sample source as described in the Methods.
Blinding	Blinding was not required for this retrospective study, which did not employ an assignment to an intervention versus control arm.

Reporting for specific materials, systems and methods

We require information from authors about some types of materials, experimental systems and methods used in many studies. Here, indicate whether each material, system or method listed is relevant to your study. If you are not sure if a list item applies to your research, read the appropriate section before selecting a response.

Materials & experimental systems

n/a	Involved in the study	
☐	☒	Antibodies
☒	☐	Eukaryotic cell lines
☒	☐	Palaeontology and archaeology
☒	☐	Animals and other organisms
☐	☒	Clinical data
☐	☒	Dual use research of concern
☒	☐	Plants

Methods

n/a	Involved in the study	
☒	☐	ChIP-seq
☒	☐	Flow cytometry
☒	☐	MRI-based neuroimaging

Antibodies

Antibodies used	Antibodies used for mass cytometry analysis include:<br><br>Mouse monoclonal CD11c (clone Bu15; Fluidigm; Cat # 3147008; RRID: AB_2687850; 3431914; 1:100), Mouse monoclonal CD123 (clone 6H6; Fluidigm; Cat # 3143014B; RRID: AB_2811081; 3431917; 1:100), Mouse monoclonal CD127 (clone A019D5; Fluidigm; Cat # 3149011; RRID: AB_2661792; 3321819; 1:100), Mouse monoclonal CD14 (clone M5E2; Fluidigm; Cat # 3151009B; RRID: AB_2810244; 2191914; 1:100), Mouse monoclonal CD15 (Clone W6D3; BioLegend; Cat # 323002; RRID: AB_756008; B254011; 1:67), Mouse monoclonal CD16 (clone B73.1; BioLegend; Cat # 360702; RRID: AB_2562693; B243320; 1:33), Mouse monoclonal CD161 (clone DX12; BD Biosciences; Cat # 556079; RRID: AB_396346; 9115548; 1:33), Mouse monoclonal CD19 (clone HIP19; Fluidigm; Cat # 3142001; RRID: AB_2651155; 3031906; 1:100), Mouse monoclonal CD1c (clone L161; BioLegend; Cat # 331501; RRID: AB_1088996; B265380; 1:100), Mouse monoclonal CD25 (clone 2A3; Fluidigm; Cat # 3169003; RRID: AB_2661806; 0342004; 1:100), Mouse monoclonal CD27 (clone L128; Fluidigm; Cat # 3167006B; RRID: AB_2811093; 2851804; 1:400), Mouse monoclonal CD279/PD1 (clone EH12.2H7; Fluidigm; Cat # 3155009B; RRID: AB_2811087; 2971910; 1:133), Mouse monoclonal CD3 (clone UCHT1; DVS Sciences; Cat # 3154003B; RRID: AB_2811086; 0071917; 1:100), Mouse monoclonal CD33 (clone WM53; BioLegend; Cat # 303402; RRID: AB_314346; B277151; 1:33), Mouse monoclonal CD34 (clone 581; Fluidigm; Cat # 3163014B; RRID: AB_2811091; 2651705; 1:33), Mouse monoclonal CD38 (clone HIT2; Fluidigm; Cat # 3172007B; RRID: AB_2756288; 0861906; 1:100), Mouse monoclonal

CD4 (clone RPA-T4; Fluidigm; Cat # 3145001; RRID: AB_2661789; 2681902; 1:100), Mouse monoclonal CD45 (Clone HI30; Fluidigm; Cat # 3089003B; RRID: AB_2661851; 2801911; 1:100), Mouse monoclonal CD45RA (clone HI100; BioLegend; Cat # 304102; RRID: AB_314406; B295482, B255475; 1:33), Mouse monoclonal CD45RO (clone UCHL1; Fluidigm; Cat # 3164007B; RRID: AB_2811092; 2431806; 1:100), Mouse monoclonal CD56 (clone N901; Fluidigm; Cat # 3176009B; RRID: AB_2811096; 3171701; 1:50), Mouse monoclonal CD7 (clone CD7-6B7; DVS Sciences; Cat # 3153014B; RRID: AB_2811084; 0282010; 1:100), Mouse monoclonal CD8a (clone RPA-T8; Fluidigm; Cat # 3162015; RRID: AB_2661802; 0171813; 1:100), Mouse monoclonal CD95 (clone DX2; BioLegend; Cat # 305602; RRID: AB_314540; B241963; 1:67), Mouse monoclonal HLA-DR (clone L243; Fluidigm; Cat # 3174001B; RRID: AB_2665397; 0991901; 1:100), Mouse monoclonal IgD (clone IA6-2; Fluidigm; Cat # 3146005B; RRID: AB_2811082; 2561908; 1:100), Mouse monoclonal IgM (clone MHM-88; BioLegend; Cat # 314502; RRID: AB_493003; B264164; 1:33), Mouse monoclonal PD-L1 (clone 29E.2A3; Fluidigm; Cat # 3156026; RRID: AB_2687855; 2761903; 1:100), Mouse monoclonal PICP (Clone PCIDG10; Millipore; Cat # MAB1913; RRID: AB_94406; 3328869, 3389939(x8); 1:133), Mouse monoclonal EMR1 (Clone BM8; BioLegend; Cat # 123102; RRID: AB_893506; B264265; 1:33), Mouse monoclonal TCR Va7.2 (Clone 3C10; BioLegend; Cat # 351702; RRID: AB_10900258; B282453; 1:33), Mouse monoclonal FOXP3 (clone 259D/C7; Fluidigm; Cat # 3159028A; RRID: AB_2811088; 1812006, 2631804; 1:50), Rabbit monoclonal phospho-4E-BP1 (Thr37 / Thr46 (clone 236B4); Cell Signaling Technology; Cat # 2855; RRID: AB_560835; 29, 31; 1:20), Rabbit monoclonal phospho-STAT1 (Tyr701 (clone 58D6); Cell Signaling Technology; Cat # 9167; RRID: AB_561284; 22; 1:400), Mouse monoclonal GZMB (clone GB11; Fluidigm; Cat # 3173006B; RRID: AB_2811095; 1611909; 1:100), Mouse monoclonal CD11b (Clone ICRF44; BioLegend; Cat # 301302; RRID: AB_314154; B286270; 1:33), Mouse monoclonal TCRgd (Clone 11F2; BioLegend; Cat # 331202; RRID: AB_1089222; B271574; 1:33), Mouse monoclonal Cleaved PARP (Clone F21-852; BD Pharmingen Customs; Cat # 624084; RRID: NA; 9326323; 1:33), Mouse monoclonal RORgt (Clone 4F3-3C8-2B7; BioLegend; Cat # 644902; RRID: AB_1595502; NA; 1:33), Mouse monoclonal T-bet (Clone 4B10; BioLegend; Cat # 644802; RRID: AB_2810251; B335065; 1:33), Mouse monoclncal CD66b (Clone G10f5; BioLegend; Cat # 305102; RRID: AB_314494; B298277; 1:308)

Validation

Mouse monoclonal CD161 (clone DX12; BD Biosciences; Cat # 556079; RRID: AB_396346), and mouse monoclonal Cleaved PARP (Clone F21-852; BD Pharmingen Customs; Cat # 624084), these antibodies were validated by flow cytometry analysis versus isotype controls. mouse monoclonal CD15 (Clone W6D3; BioLegend; Cat # 323002; RRID: AB_756008), mouse monoclonal CD16 (clone B73.1; BioLegend; Cat # 360702; RRID: AB_2562693), mouse monoclonal CD1c (clone L161; BioLegend; Cat # 331501; RRID: AB_1088996), mouse monoclonal CD33 (clone WM53; BioLegend; Cat # 303402; RRID: AB_314346), mouse monoclonal CD45RA (clone HI100; BioLegend; Cat # 304102; RRID: AB_314406), mouse monoclonal CD95 (clone DX2; BioLegend; Cat # 305602; RRID: AB_314540), mouse monoclonal IgM (clone MHM-88; BioLegend; Cat # 314502; RRID: AB_493003), mouse monoclonal EMR1 (Clone BM8; BioLegend; Cat # 123102; RRID: AB_893506), mouse monoclonal TCR Va7.2 (Clone 3C10; BioLegend; Cat # 351702; RRID: AB_10900258), mouse monoclonal CD11b (Clone ICRF44; BioLegend; Cat # 301302; RRID: AB_314154), mouse monoclonal TCRgd (Clone 11F2; BioLegend; Cat # 331202; RRID: AB_1089222), mouse monoclonal RORgt (Clone 4F3-3C8-2B7; BioLegend; Cat # 644902; RRID: AB_1595502), mouse monoclonal T-bet (Clone 4B10; BioLegend; Cat # 644802; RRID: AB_2810251), and mouse monoclncal CD66b (Clone G10f5; BioLegend; Cat # 305102; RRID: AB_314494) were quality control tested by immunofluorescent staining with flow cytometric analysis. Rabbit phospho-4E-BP1 (Thr37 / Thr46) (clone 236B4, Cat # 2855; RRID: AB_560835, Cell Signaling Technology) was validated by Western blot using 293T cells incubated for 24 hours in serum-free medium then 1 hour with amino acid deprivation, followed by treatment with amino acids with and without insulin. Rabbit phospho-STAT1 (Tyr701) (clone 58D6, Cat # 9167; RRID: AB_561284, Cell Signaling Technology) was validated by Western blot using HeLa cells treated with interferon. Mouse monoclonal CD11c (clone Bu15; Fluidigm; Cat # 3147008; RRID: AB_2687850), mouse monoclonal CD123 (clone 6H6; Fluidigm; Cat # 3143014B; RRID: AB_2811081), mouse monoclonal CD127 (clone A019D5; Fluidigm; Cat # 3149011; RRID: AB_2661792), mouse monoclonal CD14 (clone M5E2; Fluidigm; Cat # 3151009B; RRID: AB_2810244), mouse monoclonal CD19 (clone HIP19; Fluidigm; Cat # 3142001; RRID: AB_2651155), mouse monoclonal CD25 (clone 2A3; Fluidigm; Cat # 3169003; RRID: AB_2661806), mouse monoclonal CD27 (clone L128; Fluidigm; Cat # 3167006B; RRID: AB_2811093), mouse monoclonal CD279/PD1 (clone EH12.2H7; Fluidigm; Cat # 3155009B; RRID: AB_2811087), mouse monoclonal CD34 (clone 581; Fluidigm; Cat # 3163014B; RRID: AB_2811091), mouse monoclonal CD38 (clone HIT2; Fluidigm; Cat # 3172007B; RRID: AB_2756288), mouse monoclonal CD4 (clone RPA-T4; Fluidigm; Cat # 3145001; RRID: AB_2661789), mouse monoclonal CD45 (Clone HI30; Fluidigm; Cat # 3089003B; RRID: AB_2661851), mouse monoclonal CD45RO (clone UCHL1; Fluidigm; Cat # 3164007B; RRID: AB_2811092), mouse monoclonal CD56 (clone N901; Fluidigm; Cat # 3176009B; RRID: AB_2811096), mouse monoclonal CD8a (clone RPA-T8; Fluidigm; Cat # 3162015; RRID: AB_2661802), mouse monoclonal HLA-DR (clone L243; Fluidigm; Cat # 3174001B; RRID: AB_2665397), mouse monoclonal IgD (clone IA6-2; Fluidigm; Cat # 3146005B; RRID: AB_2811082), mouse monoclonal PD-L1 (clone 29E.2A3; Fluidigm; Cat # 3156026; RRID: AB_2687855), mouse monoclonal FOXP3 (clone 259D/C7; Fluidigm; Cat # 3159028A; RRID: AB_2811088), and mouse monoclonal GZMB (clone GB11; Fluidigm; Cat # 3173006B; RRID: AB_2811095) were quality-control tested by CyTOF flow cytometry analysis of stained cells using appropriate positive and negative cell staining and/or activation controls. Mouse monoclonal PICP (Clone PCIDG10; Millipore; Cat # MAB1913; RRID: AB_94406) was validated by Immunohistochemistry in human bone tissue.

Clinical data

Policy information about clinical studies

All manuscripts should comply with the ICMJE guidelines for publication of clinical research and a completed CONSORT checklist must be included with all submissions.

Clinical trial registration

https://classic.clinicaltrials.gov/ct2/show/NCT02864108

Study protocol

Research participants were enrolled to the Crnic Institute Human Trisome Project Biobank (HTP) under a study protocol approved by the Colorado Multiple Institutional Review Board (IRB; COMIRB #15-2170).

Data collection

Samples were collected through the Crnic Institute Human Trisome Project (HTP) at either the University of Colorado Anschutz Medical Campus in Aurora, Colorado, USA, or at multiple conferences in the USA.

Outcomes

The main goals of this study were 1) to define clusters or 'molecular subtypes' (MS) of individuals with trisomy 21 (T21) based on chromosome 21 (HSA21) expression data, 2) define how the multiomic landscapes of these MS differ from euploid controls (D21), and 3) how the MS differ from each other. Please see analysis section for details on whole blood RNA-seq, plasma proteomics, plasma metabolomics, plasma inflammatory markers, complete blood counts, and immune cell mass cytometry analyses.

Plants

Seed stocks	*Report on the source of all seed stocks or other plant material used. If applicable, state the seed stock centre and catalogue number. If plant specimens were collected from the field, describe the collection location, date and sampling procedures.*
Novel plant genotypes	*Describe the methods by which all novel plant genotypes were produced. This includes those generated by transgenic approaches, gene editing, chemical/radiation-based mutagenesis and hybridization. For transgenic lines, describe the transformation method, the number of independent lines analyzed and the generation upon which experiments were performed. For gene-edited lines, describe the editor used, the endogenous sequence targeted for editing, the targeting guide RNA sequence (if applicable) and how the editor was applied.*
Authentication	*Describe any authentication procedures for each seed stock used or novel genotype generated. Describe any experiments used to assess the effect of a mutation and, where applicable, how potential secondary effects (e.g. second site T-DNA insertions, mosiacism, off-target gene editing) were examined.*
